# Spatio-Temporal Coding-Based Helicopter Trajectory Planning for Pulsed Neural Membrane System

**DOI:** 10.1155/2022/1787013

**Published:** 2022-03-17

**Authors:** Jiachang Xu, Yourui Huang, Hongjin Li, Ruichong Fang, Yu Liu, Ruijuan Zhao

**Affiliations:** ^1^State Key Laboratory of Mining Response and Disaster Prevention and Control in Deep Coal Mines, Anhui University of Science and Technology, Huainan City, Anhui Province 232001, China; ^2^School of Computer Science and Engineering, Anhui University of Science and Technology, Huainan City, Anhui Province 232001, China; ^3^School of Electrical and Information Engineering, Anhui University of Science and Technology, Huainan City, Anhui Province 232001, China; ^4^School of Biomedical Engineering, Faculty of Electronic Information and Electrical Engineering, Dalian University of Technology, Dalian City, Liaoning Province 116024, China; ^5^School of Cyberspace Security, Beijing University of Posts and Telecommunications, Beijing City 100876, China; ^6^Department of Information Engineering, PLA Army Academy of Artillery and Air Defense, Hefei City, Anhui Province 230031, China

## Abstract

For the trajectory planning problem under the nonlinear and strongly coupled characteristics of unmanned helicopters, membrane computing with distributed parallel processing capability is introduced for unmanned helicopter trajectory planning. The global and local spatial information is temporally characterized; the temporal characterization algorithm under mapping information is designed; the hierarchical discriminant regression algorithm is designed based on incremental principal component analysis to realize the process of building and identifying trees in trajectory planning; and the pulsed neural membrane system (PNMS) with spatio-temporal coding function under membrane computing is constructed. Compared with the RRT algorithm in two experimental environments, the original path length, the trimmed path length, the time used to plan the trajectory, and the number of search nodes have different levels of improvement; the feasibility and effectiveness of the PNMS in unmanned helicopter trajectory planning are verified. It expands the theoretical research of membrane computing in the field of optimal control and provides theoretical support for the subsequent application practice.

## 1. Introduction

Autonomous controllability is the new trend of unmanned helicopter development, and trajectory planning is an important research content to measure the performance of helicopter autonomous controllability. Unmanned aerial vehicles (UAVs) will play an important role in the construction of smart mines, smart cities, and smart factories; the key is to solve the problems of model construction, algorithm, and scene selection. So how to effectively plan the trajectory of the helicopter flight is an important guarantee for the helicopter to complete the mission.

For the helicopter trajectory planning problem, Fu et al. used neural networks to plan the helicopter global and local paths to improve the data collection under unmanned helicopter constraints [1]. Based on the idea of guided reinforcement Q learning, Zhou et al. achieved fast convergence of the learning algorithm by continuously optimizing the path through the Q learning algorithm [2]. Mehrez and Ahmed proposed a helicopter path planning algorithms based on target trajectory regression prediction for sea surface floaters, which achieved good prediction results [3]. Lim et al. implemented unmanned helicopter path planning using heuristic angular derivatives [4]. Yang et al. elaborated various intelligent optimization algorithms for cluster path planning in path planning problem [5]. Li et al. proposed an artificial potential field fused with an ant colony algorithm for finding the optimal algorithm to avoid the localized trapping problem [6]. Yu Sheng designed a reinforcement learning-based multi-UAV path planning algorithm for different business scenarios, which improves the execution efficiency compared to traditional algorithms [7]. Zhang introduced the idea of lifting convex optimization to unmanned helicopter trajectory planning and solved this nonconvex problem by convex optimization and mixed-integer programming, thus improving the solution efficiency [8]. Zhao proposed two methods to control the saturation set and Lyapunov function to implement UAV path planning and verified the validity and reliability [9]. Wang applied expert knowledge-assisted reinforcement learning to UAV path planning by constructing potential functions through recommended trajectory order and trained by Q-learning, and the strategy got good results [10]. Roth et al. proposed a scene agent perception model and applied it to helicopter formations for perception and prediction [11]. Sun et al. proposed an edge computing framework model for the optimization of UAV trajectories, but the model was implemented based on fixed-wing aircraft [12]. Su et al. developed a trajectory optimization model for controlled variables and constraints, which overcame the algorithm's local trapping optimality but did not generalize in terms of application scenarios [13]. Farí et al. used vector fields to implement the adaptive path tracking dynamics problem in the summer without modeling, but the strategy has some room for optimization in tracking performance [14]. Park and Kim proposed a new distributed dynamic trajectory planning algorithm with the addition of constraints to improve its robustness, but the efficiency of this algorithm is 93%, which also has a lot of room for improvement [15]. The above methods undoubtedly include model construction, neural network intelligent algorithms [16], and numerical optimization to achieve good results, but the results achieved also produce problems such as possible computational enhancement and data set expansion due to restricted scenarios and error reduction; this can be further promoted and applied better in actual industrial production.

Membrane computing, as a new branch of natural computing, was proposed by Păun G of the Romanian Academy of Sciences [17, 18]. Because of its powerful parallel processing capability, it has rapidly become a hot spot for multi-disciplinary cross-research in computer, biology, control science, and artificial intelligence [19–23], and it has been theoretically studied and practically applied in multiple fields. In this paper, membrane computing is introduced to unmanned helicopter trajectory planning by constructing an impulsive neural membrane system, which can convert spatial information into temporal information by encoding spatial and temporal information, designing an incremental hierarchical discriminant regression algorithm using incremental principal component analysis, and further constructing a sample statistical model to realize unmanned helicopter trajectory planning by an impulsive neural membrane system. The main contributions of this paper are as follows:

1. A temporal representation algorithm with spatio-temporal encoding is designed with the premise of global and local spatial information representation
2. Hierarchical discriminant regression algorithm is designed to build a framework for unmanned helicopter trajectory planning, based on which tree building and recognition trees are implemented
3. The PNMS is constructed, and the effectiveness of the PNMS in trajectory planning is verified

## 2. Feature Information Extraction

For the objects of information collection, most of them are multi-feature, high-noise, and nonlinear, so information needs to be collected in the practical application process; the process of information collection is to analyze the characteristics of information. The spatial and temporal information encoding of the PNMS can convert spatial information into temporal information, and at the same time, temporal information has the characteristics of spatial information so that the encoding of time can obtain spatial information [24].

### 2.1. Global Spatial Information Representation

The global spatial information representation is rotation and translation invariant, and this feature enables autonomous navigation of the helicopter when the target position in the environment has not changed [25]. In the process of generating spatial information temporal representation by the PNMS, neurons and spatial information features possess a correspondence relationship, which can be attributed to the correspondence of neurons and feature points, and feature value inputs likewise correspond to neuron channels. The ignition neurons of each iteration of the computational process get recorded and form a time sequence over time. The elements in the sequence are the number of ignition neurons recorded for each iteration of the system, and at the same time, the sequence contains the geometric structure of spatial information, and this sequence is the global spatial information representation of spatial information.

**Figure 1:**
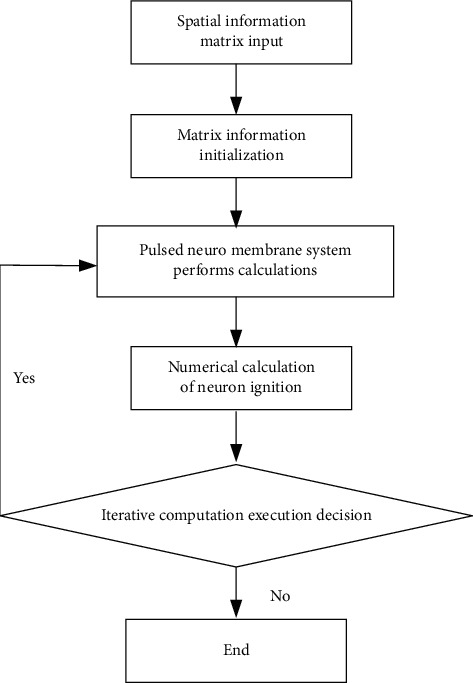
Algorithm flow for temporal representation of global spatial information.

During each iteration of the computation of the PNMS, the neuron ignition is determined jointly with the value of the feature and the issuance of the neighboring neuron pulses, and the issuance of the neighboring neuron pulses is associated with the structure of the spatial information; therefore, global temporal representation of spatial information can not only express its structure but also be used as spatial information feature. Based on the structural characteristics of neurons and their connections, the element of the global spatial information time representation of the system is the value (amount) of neuron ignition during each system iteration calculation, and the size of this value is related to the number of neurons, independent of the structure [26]. The flowchart of the global spatial information temporal representation algorithm is shown in Figure 1.

The algorithm execution process is as follows:

1. Define the input information matrix and initialize the connection matrix, the state matrix, the connection degree matrix, the threshold matrix, and the output matrix.
2. Execute the connection matrix, state matrix, and output matrix according to the predefined calculation rules.
3. Record the number of neuron ignitions. If the output matrix is satisfied, then adjust the connection degree; otherwise, continue to execute step (2).
4. Increase the number of iterative calculations while decreasing the threshold value.
5. Raise the threshold and stop the ignition after reaching the calculated value.
6. Update the number of iterations.
7. Determine whether to terminate the calculation; otherwise, continue back to step (2).

Step (3) of the algorithm in Figure 1 is used to determine whether the pulse propagation lasts in the iterative calculation process, to present the difference between the output results before and after the neuron and to improve the calculation efficiency. The time representation dimension and time after the system are executed change in the same direction.

**Figure 2:**
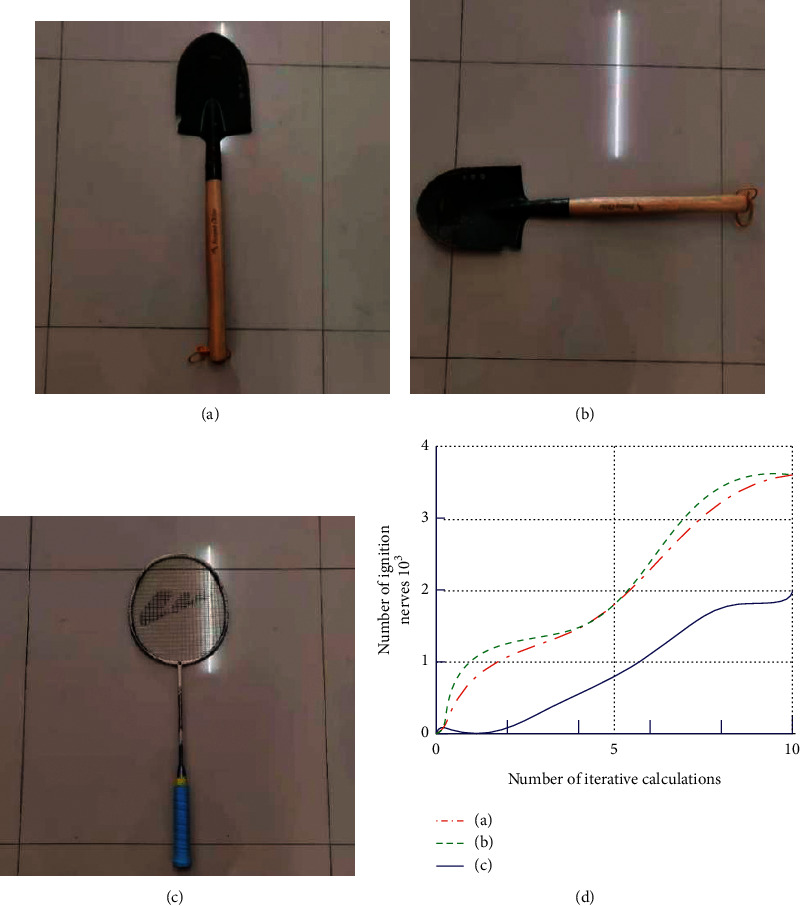
Temporal representation of global spatial information: (a) spade placed lengthwise, (b) the revolving spade, (c) badminton racket placed lengthwise, and (d) the results of pulse characterization over time.

If the spatial information is an image, the PNMS processes the spatial information in three channels to obtain a global temporal representation, as shown in Figure 2, where Figures 2(a) and 2(b) are color images under the same object, Figure 2(c) is the image after changing the object, and Figure 2(d) is the result after temporal representation. From Figure 2(d), it is shown that Figures 2(a) and 2(b) have almost the same number of ignition pulses (error neglected) under the same iterative calculation because only the same objects have been rotated, while in Figure 2(c), the results vary more because the objects in the images have changed, unlike the global temporal representation of the former.

### 2.2. Local Spatial Information Representation

From Figure 2, it can be shown that the change of spatial information cannot be expressed by the global temporal representation of the PNMS, especially during the autonomous flight of the unmanned helicopter the change of scene objects is normal, so the local spatial information representation is needed to solve this problem.

**Figure 3:**
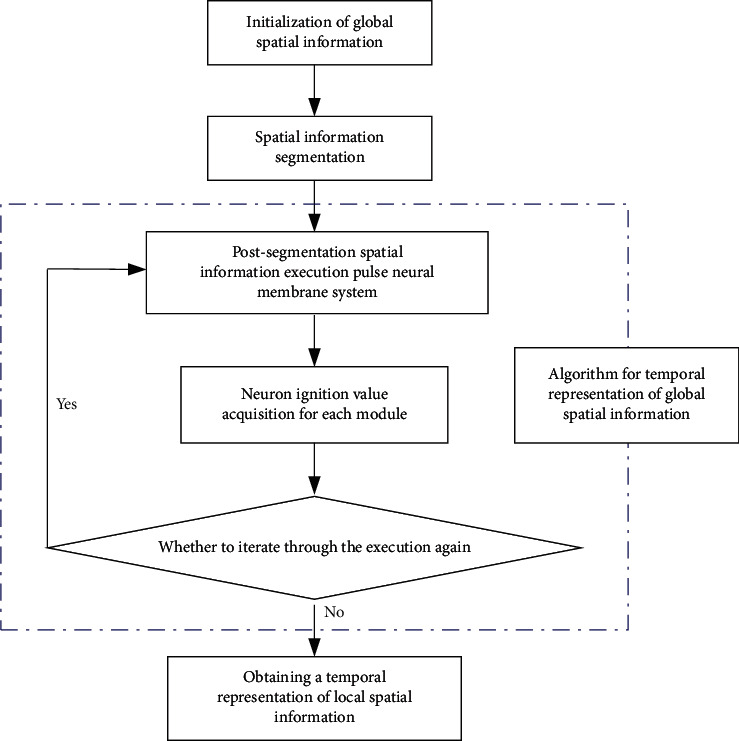
Flow of algorithm for temporal representation of local spatial information.

The local spatial information characterization process of the PNMS is to divide the original information arbitrarily according to the information characteristics and size and to carry out each independent information time characterization after the division according to the global time characterization algorithm in Figure 1. The independent information schedule collection after the characterization cooperates as the local information schedule collection, that is, to complete the local spatial information characterization under the global information characterization. The local spatial information representation algorithm is shown in Figure 3.

The local spatial information temporal representation is divided into two ways: discrete and integral. The discrete approach is to take the set of each spatial information block after division so that the pulse of the PNMS is executed in each block of the set separately, and the spatial information time representation of each division block is obtained independently. In the holistic approach, the pulses of the PNMS are executed in the whole spatial information after the division, and the pulse neural count is generated for each division block under the whole spatial information, and the temporal information representation of each division block is obtained based on the whole spatial information.

**Figure 4:**
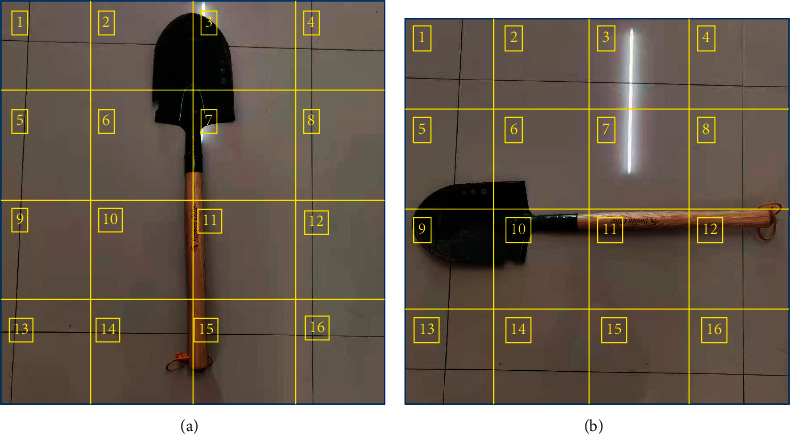
Spatial information division: (a) image information division and (b) image information partition after rotation.

In this paper, we take the overall acquisition of the divided information blocks as an example and divide the information of Figure 2 into as shown in Figure 4, assuming that the division of each block does not overlap.

**Figure 5:**
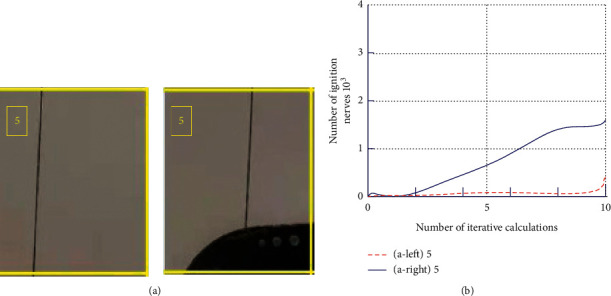
Temporal representation of local spatial information: (a) partial image information selection and (b) temporal characterization of pulse under local information.

For a clearer indication of the local spatial information temporal representation, the division block 5th in Figures 4(a) and 4(b), respectively, is taken and defined based on the local spatial information temporal representation algorithm as follows [27]:

(1) $$\begin{array}{c} \left\{\begin{array}{c} lump\left(f\left(i\right)\right)\cap lump\left(f\left(j\right)\right)=\emptyset \\ \sum_{n=1}^{N}f\left(i\right)=f \end{array}\right),\;i,j\in \left(1,2,\ldots ,N\right), \end{array}$$

where *f*(*i*) is the local information representation, *f* is the global information representation, and *lump*(*f*(*i*)) denotes the division block of *i*. The results of the local spatial information temporal representation algorithm executed under the pulsed neural membrane system are shown in Figure 5.

Combined with Figure 2 for comparison, the global spatial information temporal representation exhibits good rotation and translation invariance properties, while the temporal representation of local spatial information changes very much as shown by Figure 5, which reflects the change of local information very well; thus, the temporal representation of global and local spatial information can be well applied in helicopter target identification, tracking, or navigation performance.

## 3. Unmanned Helicopter Trajectory Planning

### 3.1. Unmanned Helicopter Trajectory Planning Process

**Figure 6:**
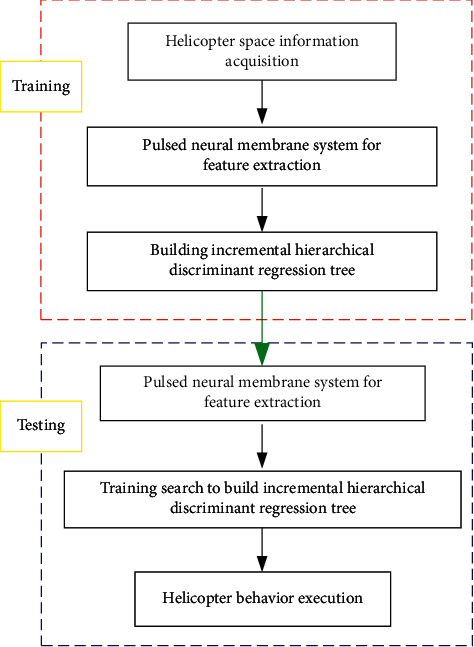
Helicopter trajectory planning process.

The helicopter trajectory planning process consists of two parts: perception and cognition [28]. The spatial information processing and feature extraction are realized by perception, and the processing results are used as input to the cognitive part, thus realizing the helicopter autonomous navigation process. In the training phase, the helicopter processes the acquired spatial information and performs feature extraction through Silan A2 LiDAR and Intel REALSENSE depth camera (integrated inertial measurement unit), that is, it acquires the temporal representation of spatial information based on the pulsed neural membrane system, uses the acquired features as input to the cognitive part in incremental principal component analysis (IPCA), and adopts incremental hierarchical discriminant regression tree building; the test phase judges the achievement of the helicopter navigation task. As can be seen from Figure 2, the global and local characteristics of the temporal representation of spatial information possess good sensitivity to the degree of spatial information variation; if the spatial information variation is small, the global spatial information temporal representation of the PNMS can be used in the helicopter perception part, and if the degree of spatial information variation is large, both global and local spatial information temporal representations can be used. The helicopter trajectory planning process is shown in Figure 6.

### 3.2. Incremental Principal Component Analysis

Principal component analysis minimizes the projection error, as a typical representative of the projection method. The projection method refers to the projection of high-dimensional data to lower dimensions, and the direction of projection can be determined by feature processing. Principal component analysis needs to incorporate all the information into the calculation; however, the method has some drawbacks for too large data sets. If the idea of dividing spatial information and selecting some information for calculation at a time is called incremental principal component analysis, this method is used not only for processing larger information but also for online processing, which is higher than principal component analysis in terms of performance.

The incremental principal component analysis is performed by spatial information principal components, computed by stepwise iterations until convergence to the desired feature vector, which can be obtained incrementally from spatial information during the fast convergence process. Assume that the set of sample vectors {*z*(*i*)} is obtained, the dimensionality is randomized as desired, and the covariance matrix *A*=*E*{*z*^*T*^(*i*)*z*(*π*)} is defined, which must satisfy *λx*=*Ax*, where *λ* is the eigenvalues. Replacing *x* with the estimate *x*(*i*) and assuming *λx*=*a*, it is obtained that [29, 30]

(2) $$\begin{array}{c} a\left(i\right)=\frac{1}{i}\sum_{j=1}^{i}z^{T}\left(i\right)z\left(i\right)x\left(\pi \right), \end{array}$$

where *a*(*i*) is the estimate of step *i*. After obtaining the estimation of *a*, the eigenvalues and eigenvectors can be obtained separately using *x*=*a*/‖*a*‖, *λ*=‖*a*‖.

In order to obtain the estimate *x*(*i*), due to the existence of *x*=*a*/‖*a*‖, consider *a*(*i* − 1)/‖*a*(*i* − 1)‖ instead of *x*(*i*) and obtain the incremental representation in the following form:

(3) $$\begin{array}{c} a\left(i\right)=\frac{1}{i}\sum_{j=1}^{i}z^{T}\left(i\right)z\left(i\right)\frac{a\left(i-1\right)}{\left\|a\left(i-1\right)\right\|}. \end{array}$$

To facilitate incremental estimation of equation (3), it can be further transformed into an iterative form as follows:

(4) $$\begin{array}{c} a\left(i\right)=\frac{i-1}{i}a\left(i-1\right)+\frac{1}{i}z^{T}\left(i\right)z\left(i\right)\frac{a\left(i-1\right)}{\left\|a\left(i-1\right)\right\|}. \end{array}$$

Here, *i* − 1/*i* is the estimated weight in the previous step; 1/*i* is the new weight, and furthermore, it is known that *a*(0)=*z*(1); *i* tends to infinity; *a*_1_(*i*)⟶±*λ*_1_*v*_1_; *v*_1_ is the eigenvector of *λ*_1_; and *λ*_1_ is the eigenvalue.

To improve the convergence speed, the forgetting factor *ε* is introduced, and equation (4) is converted to

(5) $$\begin{array}{c} a\left(i\right)=\frac{i-1-\varepsilon }{i}a\left(i-1\right)+\frac{\varepsilon +1}{i}z^{T}\left(i\right)z\left(i\right)\frac{a\left(i-1\right)}{\left\|a\left(i-1\right)\right\|}. \end{array}$$

The above process is the first order for the eigenvectors, and the higher-order vectors can be solved by the heuristic algorithm. Because of the helicopter flight characteristics and real-time calculation, the heuristic algorithm tends to cause too long solution time. According to the orthogonal characteristics of the vectors, the residuals can be added to the complementary set to calculate the relatively higher-order eigenvectors and shorten the convergence time. The execution process is as follows:

1. Initialization of the sample vector *z*(*i*)
2. Initialize the *i*-th feature vector: *a*_*n*_(*i*)=*z*_*n*_(*i*), *i*=*n*
3. Execute equation (5) when *i* ≠ *n*

When the principal component calculation is generated, the *m* maximum eigenvalue projection factors corresponding to the samples are calculated by the following equation:

(6) $$\begin{array}{c} \eta_{j}\left(i\right)=\frac{a\left(i-1\right)}{\left\|a\left(i-1\right)\right\|}z^{T}\left(i\right),j\in \left[1,m\right]. \end{array}$$

### 3.3. Incremental Hierarchical Discriminant Regression Algorithm

From the helicopter navigation process in Figure 6, the helicopter cognitive stage uses an online incremental hierarchical discriminative regression algorithm and the UAV navigation process; the training process builds a data set (cognitive tree) based on the feature information of the temporal representation of spatial information extracted by the impulse neural membrane system; and the cognitive model is used for the testing process after training, thus realizing the helicopter navigation under the incremental hierarchical discriminative algorithm.

**Figure 7:**
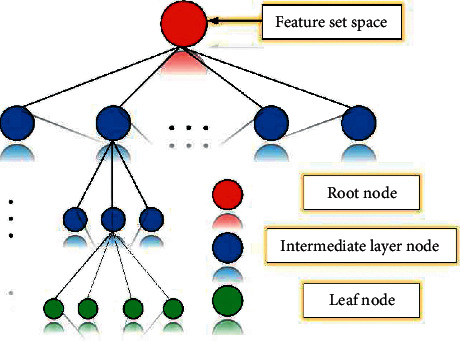
Hierarchical regression tree structure.

The cognitive model established by the hierarchical regression algorithm is characterized in the form of a tree, that is, root nodes, intermediate nodes, and leaves, as shown in Figure 7.

Hierarchical regression adopts a double clustering approach, in which the input and output clusters are computed separately at the nodes of the regression tree in accordance with the one-to-one correspondence, and the feature set (sample) data each corresponds to a vector label, and the input feature set data dimension is relatively small compared with the output dimension; meanwhile, the clusters of the input sample space generate the discriminant of the input space; and the classification sample space of each node of the regression tree is generated by the corresponding discriminant. Considering feature set of dynamics, it is difficult to differentiate with very few complete; after all, the data obtained are the current moment; subsequent data are a process of gradually reached and therefore must set a threshold value; if the current moment under the node number of the feature set is greater than the threshold, the characteristics of the node set, with the passage of time, the regression tree samples increase, continue the similar division. If the number of feature sets in the subset reaches the threshold, the subset is also divided.

**Figure 8:**
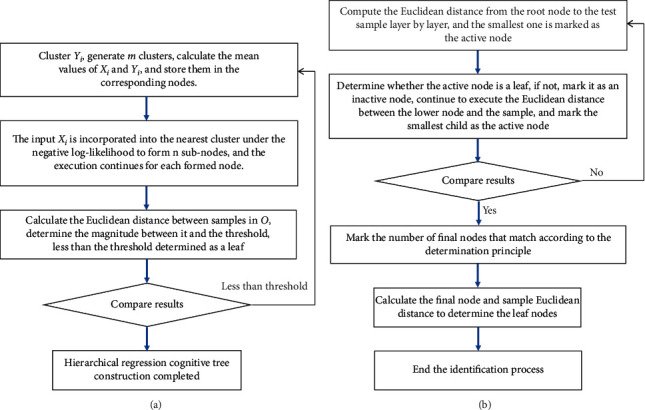
Hierarchical regression algorithm to build the tree and identify the tree: (a) tree building process and (b) identifying tree process.

Dimensionality plays an important role in the comparison process of both feature sets with subsets and subnodes including leaf nodes with sample sets. Considering the efficiency of the decision process, negative log-likelihood is established for each node so that the probability distribution can be introduced into the discriminative space of nodes, thus weakening the condition of other discriminative methods on the number of sample space and applying to the sample space under the conforming threshold, thus avoiding large samples and sample imbalance situations. In this paper, a negative log-likelihood model suitable for the threshold condition is used to satisfy the hierarchical regression algorithm with fast operational conditions. Assuming that the feature set sample space is {(*X*_*i*_, *Y*_*i*_)} and the threshold value of node output *O* is ∂_*O*_, the process of hierarchical regression algorithm to construct and identify the tree is shown in Figure 8.

Incremental hierarchical discriminant regression implements online construction of cognitive trees, which is in line with the online adaptive flight process of helicopters, by incrementally constructing cognitive trees from a large number of samples. The higher the level of the constructed tree, the smaller the variance with the input sample space cluster equation, and if the number of samples in each space is too small to satisfy the generated spatial cluster estimate, the corresponding leaf node in that space is formed. Assuming that the sample input at a moment in time axis is (*X*_*i*_, *Y*_*i*_) and the threshold is ∂_*O*_, the steps of incremental hierarchical discriminant regression to build a cognitive tree are as follows:

1. Compute the Euclidean distance between all leaves and leaf nodes of the one with the smallest value.
2. Incorporate the defined (*X*_*i*_, *Y*_*i*_) into the leaf node space computed in (1) and update the leaf node space mean value.
3. Continue to perform the Euclidean distance between the current space and all leaf spaces, and if the result appears the same as (1), that is, the distance does not change, stop the operation. If the nearest node space to *X*_*i*_ changes, (*X*_*i*_, *Y*_*i*_) is included in the changed node space, and the changed node-related values are updated, while the previous node space mean value is returned (restored).
4. When the number of samples in the leaf space is higher than a predefined threshold, build a tree, use the node as the root node, and execute in turn until the update is completed.

### 3.4. Sample Statistical Model Construction

According to the process of constructing the cognitive tree in Figure 8, we give the size of the sample space assumed at a certain small scale; given that for each node, space can have a considerable number of samples involved in the calculation, it is necessary to construct a suitable statistical model according to the actual size of the samples. Along with the existence of properties of the sample space from small to large scale, the Euclidean distance when the sample space is small, the Marxian distance when the sample space is increasing, and the Gaussian distance when it is larger are defined as follows [31, 32]:

(7) $$\begin{array}{c} L_{e}\left(X-C_{m}\right)=\ln\;{\left(2\pi \right)}^{\left(p-1/2\right)}+\ln\;{\left(\left|{Ε}_{m}\right|\right)}^{1/2}+\frac{\left(X-C_{m}\right)}{2}{\left({Ε}_{m}\right)}^{-1}{\left(X-C_{m}\right)}^{T}, \end{array}$$

(8) $$\begin{array}{c} L_{M}\left(X-C_{m}\right)=\ln\;{\left(2\pi \right)}^{\left(p-1/2\right)}+\ln\;{\left(\left|{Η}_{m}\right|\right)}^{1/2}+\frac{\left(X-C_{m}\right)}{2}{\left({Η}_{m}\right)}^{-1}{\left(X-C_{m}\right)}^{T}, \end{array}$$

(9) $$\begin{array}{c} L_{G}\left(X-C_{m}\right)=\ln\;{\left(2\pi \right)}^{\left(p-1/2\right)}+\ln\;{\left(\left|{Ν}_{m}\right|\right)}^{1/2}+\frac{\left(X-C_{m}\right)}{2}{\left({Ν}_{m}\right)}^{-1}{\left(X-C_{m}\right)}^{T}, \end{array}$$

where *C*_*m*_, Η_*m*_, and Ν_*m*_ correspond to the variance matrices.

Due to the possible variations in the size of the sample space, parametric scale factors are introduced to satisfy the process of increasing sample size, and the scale factors are defined in turn as follows:

(10) $$\begin{array}{c} \sigma_{e}=\min\left\{i,\delta_{i}\right\}, \end{array}$$

(11) $$\begin{array}{c} \sigma_{M}=\min\left\{\text{max}\left\{\text{0,}\frac{2\left(i-p\right)}{p}\right\},\delta_{i}\right\}, \end{array}$$

(12) $$\begin{array}{c} \sigma_{G}=\frac{2\left(i-p\right)}{p^{2}}, \end{array}$$

where *i* is the number of samples and *p* is the number of spaces.

Based on equations (7)–(12) defined above, the matrix weights under the number of conforming samples are introduced, and the sum of the three matrix weights is defined as follows:

(13) $$\begin{array}{c} W_{m}=W_{e}E+W_{M}H+W_{G}N. \end{array}$$

Define the normalized coefficients *σ* as the sum of *σ*_*e*_, *σ*_*M*_, and *σ*_*G*_ as 1. Then the weights in equation (13) are *W*_*e*_=*σ*_e_/*σ*, *W*_*M*_=*σ*_M_/*σ*, and *W*_*G*_=*σ*_G_/*σ*, respectively. According to equation 13, the negative logarithmic likelihood distance of the m-th input node is as follows:

(14) $$\begin{array}{c} L\left(X-C_{m}\right)=\ln\;{\left(2\pi \right)}^{p-1/2}+\ln\;{\left(\left|W_{m}\right|\right)}^{1/2}+\frac{\left(X-C_{m}\right)}{2}{\left(W_{m}\right)}^{-1}{\left(X-C_{m}\right)}^{T}. \end{array}$$

## 4. Construction of Spatio-Temporally Encoded Pulsed Neural Membrane System

The PNMS consists of neurons (single cells), a large number of which form a directed graph. Neurons send pulses to neighboring neurons through synapses, and the pulses undergo transformation (evolution) through excitation rules [33, 34]. The PNMS with a definition of degree *n* is as follows:

(15) $$\begin{array}{c} \Pi =\left(O,\sigma_{1},\sigma_{2},\ldots ,\sigma_{n},syn,f_{w},f_{L},in,out\right). \end{array}$$

where

(1) *O*={*p*} denotes a single letter set and *p* represents a pulse
(2) *σ*_1_, *σ*_2_, ···, *σ*_*n*_ denotes the *n* neurons in the system, each neuron *σ*_*i*_=(*m*_*i*_, *R*_*i*_), 1 ≤ *i* ≤ *n*, where:
  (1) *m*_*i*_ is the number of pulses contained in neuron *σ*_*i*_ in the initial state of the system calculation.
  (2) *R*_*i*_ denotes the set of all rules in neuron *σ*_*i*_. The rule representation is as follows:
    ① *E*/*a*^*x*^ → *a*^*y*^; *q*. *E* denotes regular expressions of *a*, 1 ≤ *y* ≤ *x*, *q* ≥ 0, and this type is an excitation rule
    ② *p*^*r*^ → *λ*, *r* ≥ 1, *R*_*i*_ belongs to type ① of *E*/*a*^*x*^ → *a*^*y*^; *q*; there exists *p*^*r*^ ∈ *L*(*E*); *L*(*E*) is the language represented by the regular expression *E*; and this type is the forgetting rule
(3) *syn*⊆{1,2, ···, *n*} × {1,2, ···, *n*} denotes the connections between neurons, (*i*, *i*) ∉ *syn*.
(4) *f*_*w*_ represents the weight function between neurons at time *t*. At the current moment, if the neuron is “active,” the neuron will send a certain number of pulses to the neighboring neurons, and the number of pulses received by the receiving neuron is determined by the weight function of the two neurons; conversely, if the neuron is “inactive,” the neighboring neuron will not receive pulses, and the pulses will be output to the system according to the rules.
(5) *f*_*L*_ denotes the test function of the PNMS, which together with *f*_*w*_ maps the weight function at the next moment, according to *f*_*w*_(*t*) at time *t*, and characterization of *f*_*w*_(*t*+1) by *f*_*L*_(*t*).
(6) *in*, *out* ∈ {1,2, ···, *n*} denotes input and output.

**Figure 9:**
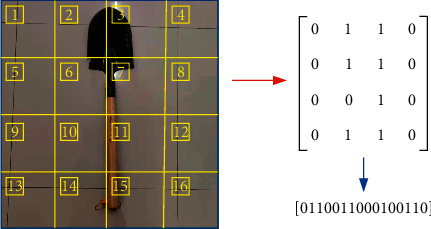
Pulse string conversion diagram.

Since the PNMS can only input pulse strings, the information needs to be characterized in the form of strings. According to the characteristic information representation method in this paper, the segmentation process of information is converted into a sequence string, and the division of information 4*∗*4 is changed into the form of matrix 4*∗*4 as shown in Figure 9 as an example in Figure 4(a). The converted binary string consisting of 0 and 1 can be used as the input of the system.

From equation (15), the input process of the system is carried out by a neuron that has a specialized input synapse, that is, it acquires information from the environment. The input neuron acquires a 16 bit binary string, and if it reads a “1” in the current string, it acquires a pulse, and vice versa; it does not acquire a pulse; and 16 units (moments) are read. If the neuron is “active” after receiving a pulse, it sends a pulse to the neighboring neuron if there is a “0” in the current string, it means that there is no pulse input at the current moment and the neuron cannot be “active” at the next moment.

In the training process, the number of neurons in the 4*∗*4 number is determined by the specific “value” of the input string, which ultimately determines the specific number of pulses a neuron contains. In this process, the neuron does not execute the forgetting statute; the neuron only makes the decision to send or not to send pulses to neighboring neurons according to the “active” and “inactive” states. Based on the change of the weight function in the above-defined system, the synaptic weights between neurons change with the passage of time (moments). All neurons follow this rule to calculate the final value of intersynaptic weights over time due to the presence or absence of pulse transmission.

**Figure 10:**
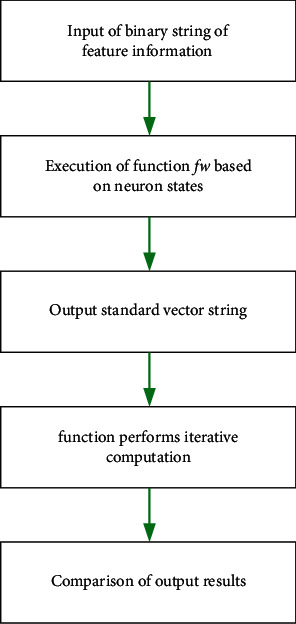
Test computing flow.

The test process defines the pulse generation process of the output neurons in the training phase to form the representation vector parameters in accordance with the helicopter control, and this process finally results in the output vector of the system. The computational flow of the test process is shown in Figure 10.

## 5. Experimental Verification

The experiments in this paper are implemented based on MeCoSim and MATLAB environment, combined with the unmanned helicopter comprehensive experimental platform built by the authors of this paper [35]. To verify the feasibility and effectiveness of the PNMS, two different experimental scenarios are built, each with a width of 5.8 m and 3 m, respectively, and the starting and ending points are set in the experimental scenarios, with red dots representing the starting points and green squares representing the ending points.

**Figure 11:**
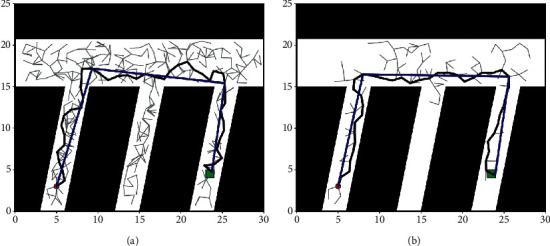
Experimental results obtained for the first scenario: (a) experimental results of RRT algorithm and (b) experimental results of PNMS.

**Figure 12:**
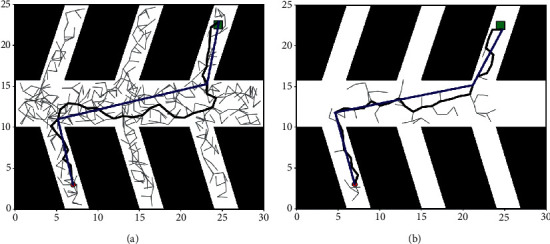
Experimental results obtained for the second scenario: (a) experimental results of RRT algorithm and (b) experimental results of PNMS.

In order to achieve comparability of the experimental results, the classical RRT algorithm was selected for comparison. The reason why the RRT algorithm was selected is that it is more mature in robot path planning or UAV trajectory planning applications [36]. The results obtained from the experiments in the two scenarios are shown in Figures 11 and 12.

Experiments are performed using the classical RRT algorithm in Figures 11(a) and 12(a), and the temporally encoded impulsive neural membrane system algorithm proposed in this paper is used in Figures 11(b) and 12(b). The gray line segment in the figure shows the tree branch searched by the algorithm; the black line segment shows the path searched by the algorithm; and the blue line segment shows the pruned path. It can be seen from the figure that in two different experimental scenarios, the classical RRT algorithm needs to perform a large number of searches and generate a large number of nodes, and the smoothing of the searched trajectory is poor and the length of the trajectory is large, indicating that the PNMS algorithm proposed in this paper can search well in narrow paths, the tree branches elongate in the direction of the target with a certain probability. The number of nodes used to search for the path is significantly reduced; the trajectory planning is relatively smooth; and the trajectory length of the planned path is relatively small. The detailed data obtained from the experiments are shown in Table 1.

From Table 1, it can be seen that the method proposed in this paper in environments 1 and 2; planning the original path length is 6.98% and 11.11% shorter than the RRT algorithm, respectively; the trimmed path length is 3.94% and 2.06% shorter, respectively; the time used to plan the trajectory is reduced by 66.67% and 86.30%, respectively; and the number of search nodes used to plan the path is reduced by 66.23% and 84.28%, respectively. The above shows that the improved algorithm has a great improvement in planning efficiency relative to the original algorithm, which is suitable for helicopter trajectory planning in a coal mine tunnel.

## 6. Conclusions

In this paper, membrane computation is introduced to an unmanned helicopter trajectory planning by using unmanned helicopter as a carrier and obtained the following conclusions:

1. The spatio-temporal encoding with the global and local spatial information representation is feasible to introduce unmanned helicopter trajectory planning
2. Unmanned helicopter trajectory planning process is reasonable, and it is proved theoretically
3. The experimental results obtained ideal results in terms of planning path and pruning path, planning time, and search nodes number compared with the classical RRT algorithm, which proved that the PNMS has good feasibility and effectiveness in unmanned helicopter trajectory planning

The follow-up work, based on the results of this paper, we continue to do research on the optimization of PNMS construction and strive to put the theoretical research results into practice.

## Figures and Tables

**Table 1:** Comparison of track planning data of two algorithms.

Experimental scenario	Track planning algorithm	Original path length	Trim path length	Planning time	Number of search nodes
Scenario 1	Classic RRT	51.60	43.44	0.45	382
	PNMS	48.00	41.73	0.15	129

Scenario 2	Classic RRT	43.20	34.44	0.73	509
	PNMS	38.40	33.73	0.10	80

## Data Availability

The experimental data and program code can be obtained from the authors of this paper.
